# A Novel Mobile Tool (Somatomap) to Assess Body Image Perception Pilot Tested With Fashion Models and Nonmodels: Cross-Sectional Study

**DOI:** 10.2196/14115

**Published:** 2019-10-29

**Authors:** Christina Ralph-Nearman, Armen C Arevian, Maria Puhl, Rajay Kumar, Diane Villaroman, Nanthia Suthana, Jamie D Feusner, Sahib S Khalsa

**Affiliations:** 1 Laureate Institute for Brain Research Tulsa, OK United States; 2 School of Psychology University Park University of Nottingham Nottingham United Kingdom; 3 Jane and Terry Semel Institute for Neuroscience and Human Behavior University of California Los Angeles Los Angeles, CA United States; 4 International Technology Consulting, LLC Los Angeles, CA United States; 5 Department of Neurosurgery University of California Los Angeles Los Angeles, CA United States; 6 Oxley College of Heath Sciences University of Tulsa Tulsa, OK United States

**Keywords:** body image, body perception, body representation, body image disorder, eating disorder, mobile health, mental health, mobile app, digital health

## Abstract

**Background:**

Distorted perception of one’s body and appearance, in general, is a core feature of several psychiatric disorders including anorexia nervosa and body dysmorphic disorder and is operative to varying degrees in nonclinical populations. Yet, body image perception is challenging to assess, given its subjective nature and variety of manifestations. The currently available methods have several limitations including restricted ability to assess perceptions of specific body areas. To address these limitations, we created Somatomap, a mobile tool that enables individuals to visually represent their perception of body-part sizes and shapes as well as areas of body concerns and record the emotional valence of concerns.

**Objective:**

This study aimed to develop and pilot test the feasibility of a novel mobile tool for assessing 2D and 3D body image perception.

**Methods:**

We developed a mobile 2D tool consisting of a manikin figure on which participants outline areas of body concern and indicate the nature, intensity, and emotional valence of the concern. We also developed a mobile 3D tool consisting of an avatar on which participants select individual body parts and use sliders to manipulate their size and shape. The tool was pilot tested on 103 women: 65 professional fashion models, a group disproportionately exposed to their own visual appearance, and 38 nonmodels from the general population. Acceptability was assessed via a usability rating scale. To identify areas of body concern in 2D, topographical body maps were created by combining assessments across individuals. Statistical body maps of group differences in body concern were subsequently calculated using the formula for proportional z-score. To identify areas of body concern in 3D, participants’ subjective estimates from the 3D avatar were compared to corresponding measurements of their actual body parts. Discrepancy scores were calculated based on the difference between the perceived and actual body parts and evaluated using multivariate analysis of covariance.

**Results:**

Statistical body maps revealed different areas of body concern between models (more frequently about thighs and buttocks) and nonmodels (more frequently about abdomen/waist). Models were more accurate at estimating their overall body size, whereas nonmodels tended to underestimate the size of individual body parts, showing greater discrepancy scores for bust, biceps, waist, hips, and calves but not shoulders and thighs. Models and nonmodels reported high ease-of-use scores (8.4/10 and 8.5/10, respectively), and the resulting 3D avatar closely resembled their actual body (72.7% and 75.2%, respectively).

**Conclusions:**

These pilot results suggest that Somatomap is feasible to use and offers new opportunities for assessment of body image perception in mobile settings. Although further testing is needed to determine the applicability of this approach to other populations, Somatomap provides unique insight into how humans perceive and represent the visual characteristics of their body.

## Introduction

Accurately perceiving the overall state of the body is a key sensory task necessary for health maintenance in humans [1] and can be subdivided into two domains: (1) interoception, the process by which the brain senses and perceives internal body signals such as the feeling of one’s heartbeat, breath, or intestines [2], and (2) exteroception, the process by which the brain senses and perceives external body signals, such as the sight, sound, shape, or texture of an object [3]. Clinicians rely on patients to have an accurate translation from sensation to perception during diagnostic assessment of medical and psychiatric symptoms and treatment selection and delivery. However, in certain cases, perceptual inaccuracy (ie, discrepancies between the person’s receipt of body signals and his/her corresponding interpretation) is an important diagnostic characteristic contributing to the expression of mental health disorders, for example, perceived physical flaws in body dysmorphic disorder, body image disturbance in eating disorders, and distressing body sensations in somatic symptom disorders [4]. Adequately characterizing these body misperceptions poses a significant challenge in mental health settings.

Body dissatisfaction, defined as unhappiness with self-perceived flaws in body features, is an especially common issue for women [5], particularly negative body attitudes that are often related to perceptions of the visual appearance of body regions such as the abdomen, hips, and thighs [6]. Self-discrepancy theory, in relation to body dissatisfaction, proposes that negative feelings and thoughts toward oneself stem from disparities between the size/weight/shape of individuals’ current versus their ideally desired body figure. Therefore, body dissatisfaction is often measured by the difference between an individual’s perceived current body figure and their body figure ideal, utilizing a menu of standardized body silhouettes to choose from. Body dissatisfaction assessed in this way has been shown to be significantly associated with symptoms of eating disorders [7-9] and other psychiatric conditions such as depression [10]. Despite these findings, a failure to identify the specific negative thoughts and feelings associated with body dissatisfaction might result in an incomplete picture of body perception. Additionally, there are non–weight-related body characteristics that are not typically included in standard measurements of body dissatisfaction including perceived abnormalities of excessive sweating, emitted odors, shape of facial features, and skin condition.

Disturbances of body perception often occur in individuals with psychiatric disorders. For instance, individuals with anorexia nervosa tend to overestimate characteristics of certain body areas relative to healthy comparisons [11] and may perceive body parts such as their waist, hips, bust, and face as much larger than they actually are, even when emaciated [12,13]. This form of body image disturbance is a core diagnostic feature of the disorder [4], a significant predictor of relapse [14], and an indicator of poor outcome [14-16]. Misperceptions of appearance are also a core feature of body dysmorphic disorder, a psychiatric condition that affects men and women in nearly equal proportions and commonly co-occurs with anorexia nervosa [4,17-19]. Disturbances in perception of body size and shape in these clinical populations have been associated with specific neurobiological signatures, providing initial insights into the pathophysiology of abnormal body image perception. For example, several studies have linked body image disturbance to abnormal functioning in cortical visual systems in anorexia nervosa and body dysmorphic disorder [20-22]. Moreover, when viewing their own bodies, individuals with anorexia nervosa display abnormal activity in visuospatial processing regions such as the inferior parietal lobule and precuneus [23-27] as well as the occipitotemporal cortex (including extrastriate body area) [28]. When viewing others’ bodies, individuals with anorexia nervosa demonstrate increased activation of the superior parietal lobule, inferior and middle frontal gyri, thalamus [24], and amygdala [26]. Weaker connectivity between precuneus and midtemporal regions when viewing others’ bodies has also been observed in anorexia nervosa [29]. In addition, weaker connectivity from the left fusiform body area to the left extrastriate body area has been associated with increased body size misjudgment in anorexia nervosa [30]. Thus, in both anorexia nervosa and body dysmorphic disorder, there is evidence of disturbances in extended body processing networks, including visual sensory systems, that may be associated with perceptual abnormalities and contribute to abnormal body perception.

It is less clear if and how nonclinical populations differ in body image perceptions. Discordant body perceptions (eg, body dissatisfaction or seeing one’s self as “fat” when slim) have been theorized to be strengthened and intensified for some women by social media and media image exposure [31-33]. Fashion models, and other models, are disproportionately exposed to both social media and media image focus on their own bodies. They are selected for this occupation largely on the basis of their physical appearance, and they regularly receive explicit feedback on the details of their visual appearance. Fashion models also experience frequent measurements of body parameters that can be associated with critiques about their body, both in terms of modifiable characteristics such as weight and body shape and unmodifiable characteristics such as height. It is unclear if membership in this group is associated with enhanced or altered perceptual accuracy for the human body overall, for specific body areas, and heightened emotional responses to body concerns.

Most current body perception assessments rely on language-based methods such as verbal interviews and questionnaires. Verbal interviews typically involve an in-person discussion with a clinician or researcher, which is time intensive, requires specialized training, and may lack the degree of specificity needed for capturing an accurate snapshot of body-related perceptions or concerns. For example, it can be challenging to describe in words exactly how large one perceives a particular area of their body to be. Questionnaire-based scales measuring body image perceptions typically assess attitudes about the body, both negative [34] and positive [35]. However, there is still a need for body image assessments that include the perceptual details about individual body concerns, emotions, distress, or specific body areas (eg, stomach, thighs, and bust). One way of assessing the perceptual details of body image is to use visually based tools. The most commonly employed methods use still photographs [5] or 2D drawings of a silhouetted figure, for example, the Stunkard Figure Rating Scale [36], which depicts several different-sized versions of a basic body outline. The individual selects the body figure that overall best represents how they perceive their current and ideal body size/shape. Unfortunately, these methods obscure considerable body details, rendering, at best, a gestalt proxy for whole body perception. Computer-based tools have been previously created as an additional or alternative to questionnaires and visually based assessments [37-39], and more recently, have utilized 3D avatars that can be manipulated by individuals [40]. However, these tools in their current format may also have certain limitations, including a reduced ability to manipulate different body areas independently (such as the width or length of body parts) and they may lack assessments of non–size-related perceptual concerns (eg, perspiration, body odor, or a skin condition). In addition, none of the aforementioned tools are deployed on mobile devices, which may be important in facilitating longitudinal, home-based assessment and tracking clinical trajectory [41].

To address existing gaps in the ability to accurately assess body perceptions, we developed Somatomap, a novel mobile tool intended to quantitatively and qualitatively assess different aspects of body image perception in 2D (ie, mapping body concern, types of concern, and emotions associated with concern) and 3D (ie, measuring the degree of disturbance of body image perception for body part sizes and shapes). In this manuscript, we describe the development of this tool for assessing body image perception and results of pilot feasibility and usability testing in female fashion models and in a general population reference sample. Given the greater attention and feedback applied to their own visual body characteristics as a function of their occupation, we hypothesized that fashion models might (1) perceive concerns with areas of the body that distinctly differ from nonmodels, and (2) that they would be more accurate in estimating the size of their body parts and overall body size. Finally, we predicted that the Somatomap tool would be sensitive to detecting both kinds of differences.

## Methods

### Somatomap

**Figure 1:**
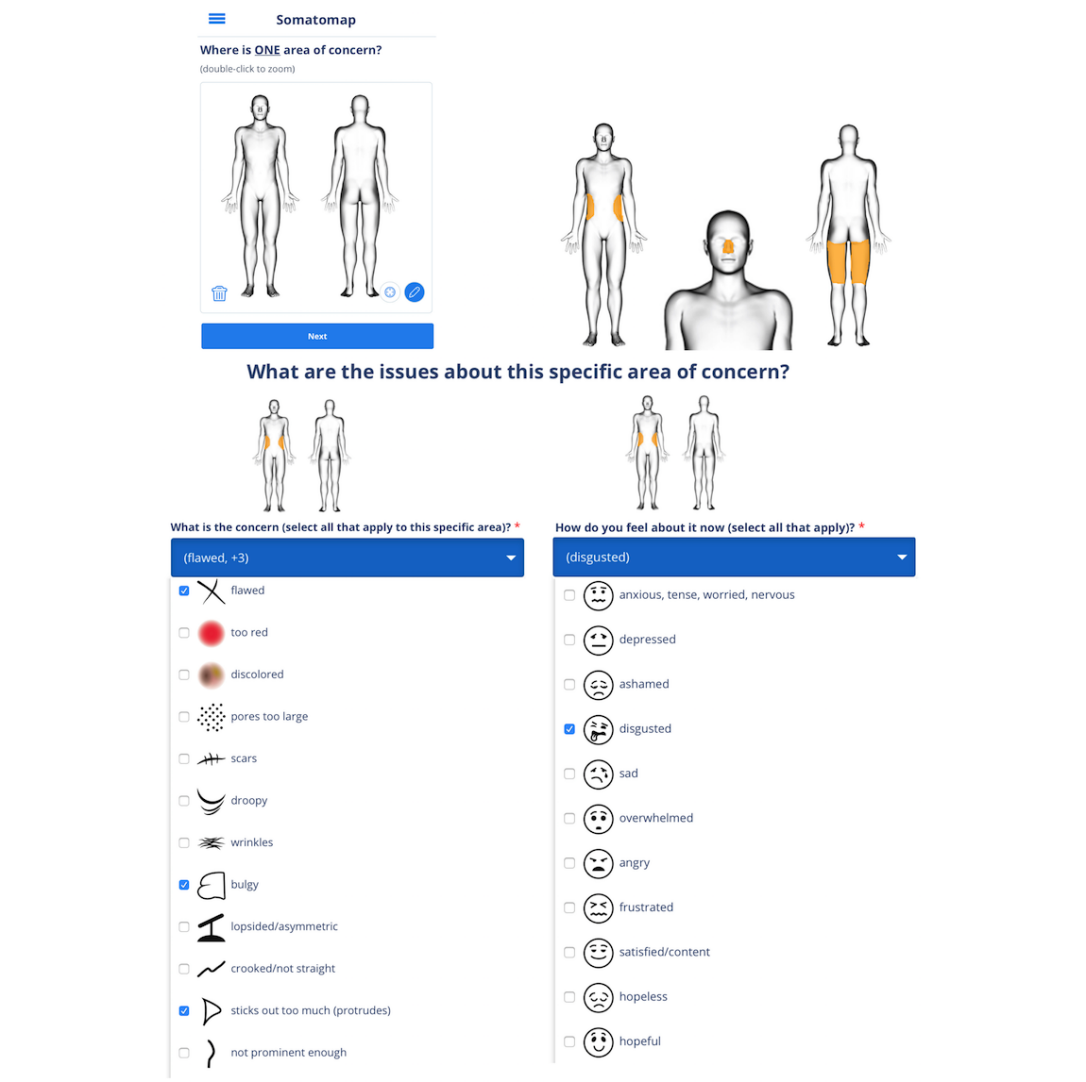
Somatomap 2D. Step-by-step screenshots of avatars and a subsample of possible body concerns and emotion ratings that can be endorsed for the 2D assessment. Participants first indicate one area of body concern by outlining it on the avatar (top left and top right), with the ability to zoom in by double tapping the figure to indicate body concerns for smaller areas or areas with more detail (top right). They are then asked to select the type of concerns pertaining to the body area (bottom left shows a subsample with several concerns selected; users can also enter a unique concern if theirs is not listed). Finally, they are asked to choose the feelings pertaining to the area of body concern (bottom right shows a subsample) or enter their own feelings. Participants then repeat this process for each body concern. The top right depicts three different examples of body concern outlines.

**Figure 2:**
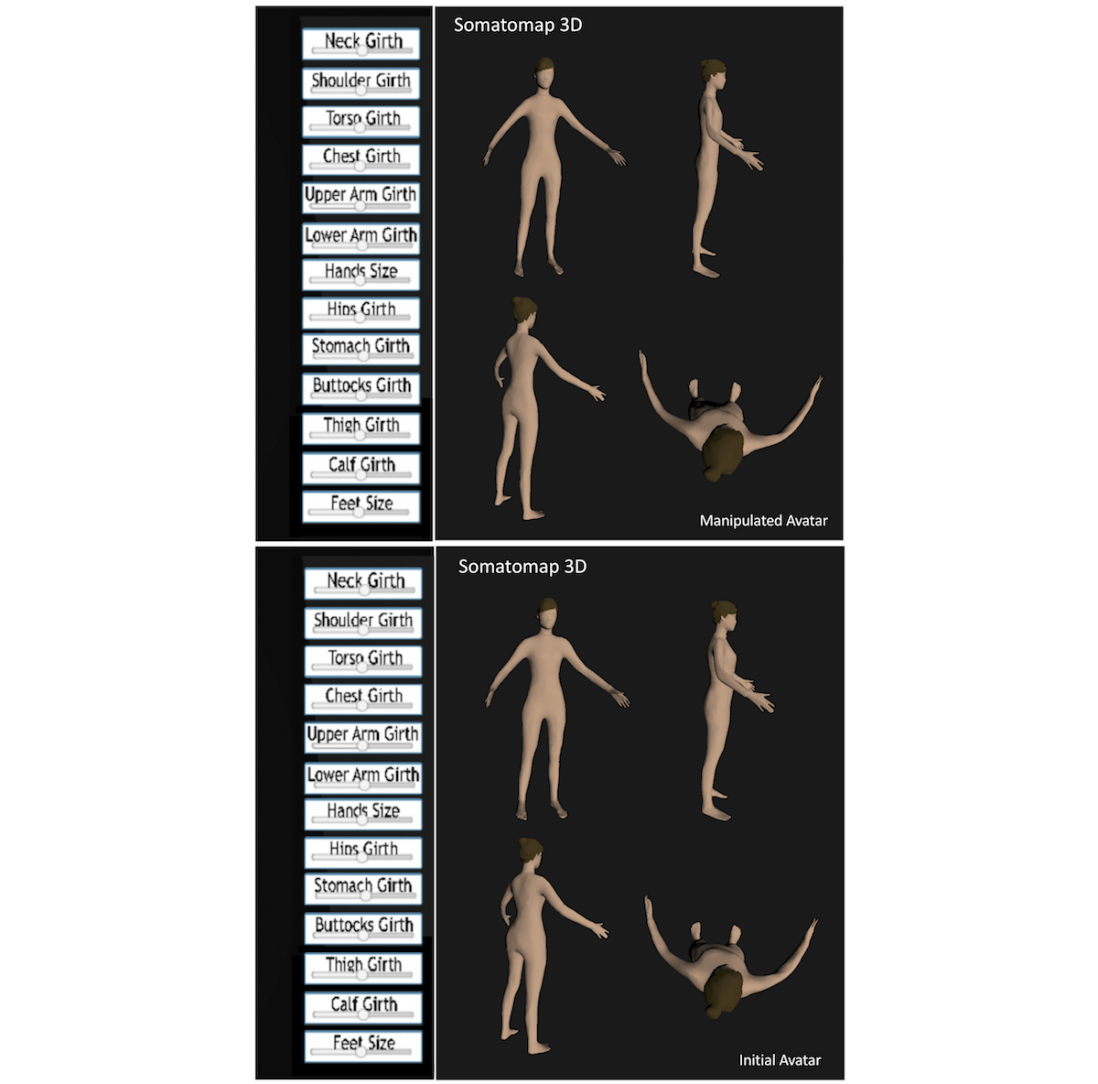
Somatomap 3D. Step-by-step screenshots of avatars for the 3D assessment. Bottom: 3D avatar shown at the start of the assessment. Participants were instructed as follows: “Please use the sliders at the left to create what your body looks like today.” Participants could rotate the avatar to view it from multiple angles as they manipulated the sliders (screenshots show examples of different orientations). Only a single avatar is visible at any given time. Top: Example of a final avatar after manipulating the sliders (shown from multiple angles matching the original avatar).

We developed Somatomap as a Web-based self-assessment tool for measuring body image perception in 2D and 3D. The 2D assessment displays a picture of an androgynous manikin; the user is asked to imagine this manikin as their own body and draw directly upon it to outline an area where they perceive a body concern (Figure 1). We used an androgynous manikin to obviate the need for spatial normalization or registration when performing statistical comparisons across individuals with different body sizes and shapes (eg, male/female or obese/slender). Users subsequently answer questions detailing specific characteristics of this concern. To capture emotional experiences that are often related to appearance concerns, they provide associated emotion ratings (eg, by selecting face emotion icons with associated labels such as “sad,” “disgusted,” “ok/fine,” “other [please be specific]”). Visual icons/emoticons accompany written labels to help illustrate the different types of perceptual and affective experiences a user might experience. The process is repeated separately for each concern. The 3D assessment displays a virtual avatar in 3D, enabling participants to rotate and view it from different angles; the user is asked to imagine the avatar as their own body and to adjust the skin and hair color as well as the size of individual body parts to reflect the perceived characteristics of their current body (Figure 2). The 3D avatars were created with 3D scans from a male and a female human volunteer using a 3D camera (Eva Lite Scanner, Artec Inc, Santa Clara, CA) and 3D software (Studio 11 Professional, Artec Inc) to create a male and female 3D mesh. These 3D meshes were imported individually into modeling software (Maya, Autodesk Inc, San Rafael, CA) to add modification ability. Within Maya, each 3D scan was cleaned into a fully smoothed mesh. The faces were altered for anonymity and to facilitate identification with a generic avatar. We selected 13 different regions of the body to modify independently: neck, shoulders, torso, bust, bicep, forearm, hands, hips, waist, buttocks, thighs, calves, and feet. Each region was modified into a maximum and minimum version using the blend shape functionality of the modeling software. The resulting Unity 3D Web plugin was uploaded into the Chorus platform. Using the Web-based display, participants could view the avatar from all angles, manually select each body area, and then use a horizontal slider control to adjust the size of the area (moving between two extreme body sizes). Importantly, each body area could be adjusted independent of the others, allowing for a variety of combinations.

Somatomap was built on Chorus, a HIPAA (Health Insurance Portability and Accountability Act)-compliant visual development platform for creating mobile Web, text-messaging, and interactive voice apps [42]. Chorus is a hosted service provided through the University of California Los Angeles [42]. Chorus apps including Somatomap are compatible with mobile phones, tablets, and desktops that have access to major Web browsers (such as Google Chrome, Apple Safari, and Firefox). This mobile compatibility enables users to complete assessments at home with devices they already have. All data are encrypted between devices and the centralized server.

### Participants

We recruited a sample of 65 female fashion models (age=23.4 [SD 5.5] years) from professional modeling agencies in the United Kingdom. Models were initially recruited telephonically and asked to visit their agency; all who were contacted came in. We also recruited a sample of nonmodels (n=38; age=25.4 [SD5.2] years) from the general UK population through flyers and social media. Neither group was informed about the study hypotheses in advance of the study, and none declined to participate after arriving for the consenting procedure and evaluation in either group.

### Data Collection

The study was approved by the School of Psychology Ethics Review Board at the University of Nottingham. Testing sessions occurred for fashion models at their modeling agencies and for nonmodels at the University of Nottingham. Prior to the experiment, each participant provided written informed consent. Participants were seated at a laptop computer to complete demographic questions adapted from the PhenX toolkit [43], and three assessments (Somatomap 2D, Somatomap 3D, and 3D usability assessment) were presented on a laptop using the Chorus [42] platform. The order in which Somatomap 2D and Somatomap 3D were presented was counterbalanced.

In Somatomap 2D, participants were asked to outline a specific area of body concern on a 2D human manikin using a laptop trackpad (13-inch MacBook Air, Apple Inc). Once the outline is drawn, the interior automatically fills in, resulting in an “area of concern.” This procedure gave participants maximum flexibility to trace any body region they chose, with pixel-level specificity. They then entered details about their concern by selecting each type of concern and the emotions surrounding the concern and used a slider to indicate the magnitude of the body concern. If they had more than one body concern, they repeated this procedure for each individual area of concern.

In Somatomap 3D, participants could rotate a 3D human avatar in multiple directions and adjust body areas independent from one another. Participants were instructed as follows: “Please use the sliders at the left to create what your body looks like today.” The 3D usability assessment was an online questionnaire asking about their experience of using the app. Questions asked how difficult/easy and frustrating/enjoyable the tool was to use and assessed the degree of identification with the original avatar (before moving the sliders) and the final avatar (after completing moving the sliders).

After completing all body image perception ratings, each participant’s shoulders, bust, biceps, waist, hips, thighs, and calves were measured with a tape measure following a standardized protocol adapted from the PhenX toolkit [43]. Each participant’s actual body mass index (BMI) was calculated by using a stadiometer for height and a bioimpedance scale (Tanita Inc) for weight. The entire study, including the consent, physical measurements, and debrief, took approximately 30 minutes.

### Statistical Analysis

#### Somatomap 2D

Proportional maps of body concern for each group were generated from Somatomap 2D tracings by collapsing across all areas of body concerns. This approach to proportionally display body concerns is similar to our previously published studies involving body maps of cardiac sensation [44-46]. Each pixel in the proportional body map thus represented the proportion of participants reporting some type of concern in that area of the body. To statistically evaluate between-group differences in body concern, we calculated a proportional z-score statistic for each pixel, following our previous method [46]. To estimate the *P* value for the calculated z-value, we utilized permutation testing, a statistical resampling method. Permutation testing assumes that under the null hypothesis, the group labeling of participants (model or nonmodel) is arbitrary and that one can estimate the probability distribution of the test statistic under the null hypothesis by randomly relabeling participants and computing the test statistic. We used 5000 permutations, similar to our previous study [46]. To calculate *P* values for each pixel, we compared the *z*-value from the actual sample to the number of occurrences of a *z*-value in the resampled set that were equal or larger to the true *z*-value. Maps were cluster corrected and spatially smoothed using a Gaussian kernel with full width half maximum of 6 pixels, and pixels with *P* values<.05 were considered significant [46]. 

#### Somatomap 3D

Perceived body measurement values were converted from arbitrary units to centimeter units via piecewise linear interpolation, using the actual body part sizes of the initial female volunteer who was scanned to create the 3D avatar. Body parts were measured using an in-engine ruler for three situations: when the slider was set to 0.5, when it was set to 0, and when it was set to 1. Separate linear interpolations for values between 0 and 0.5 and for values between 0.5 and 1 were computed. Premeasured values for the 0.5 setting allowed for calculations of the appropriate scale factor by multiplying by the amount of relative change for each part computed earlier. For example, “0” on the slider might actually mean the foot is 75% of its size when the slider is at “0.5,” and “1” on the slider might mean the foot is 130% of its size when the slider is at “0.5.” Such measurements and calculations were performed independently for each model and their constituent body parts. Discrepancy scores (in centimeters) were then calculated by subtracting the actual body measurement from the perceived body measurement for each of the seven body areas physically measured.

A multivariate analysis of covariance was used to determine if there were group differences in the actual body measurements, the 3D body measurements, and the discrepancy scores. Covariates included BMI, height, and weight. If the multivariate analysis of covariance results were significant, post hoc analysis using analysis of covariance was used to determine which specific variables showed differences between models and nonmodels.

## Results

### Participant Demographics

Key demographic data are included in Tables 1 and 2. We performed *t* test comparisons and Chi-square statistics between model and nonmodel participants to determine any significant differences between the two groups. There was no significant difference for overall race/ethnicity (*χ^2^*_4_=4.9; *P*=.29), and family income was comparable between models and nonmodels (t_88_=1.07, *P*=.29). Models had a significantly lower BMI (*P*<.001), which was driven by differences in height (*P*<.001) but not body weight (*P*=.69). Nonmodels also reported completing significantly higher levels of education than the models (*χ^2^*_5_=41.1; *P*<.001).

**Table 1:** Demographic characteristics of female fashion models (n=65) and nonmodels (n=38) analyzed by t test.

Characteristics	Model, mean (SD)	Nonmodel, mean (SD)	*t* (*df*)	*P* value
Age (years)	25.4 (5.2)	23.4 (5.5)	1.7 (80.9)	.09
Height (cm)	175.9 (5.1)	162.5 (6.3)	–11.2 (65.3)	<.001
Weight (kg)	57.5 (4.4)	56.9 (8.4)	–0.4 (48.7)	.69
Body mass index (kg/m^2^)	18.6 (1.2)	21.3 (2.8)	5.7 (44.2)	<.001

**Table 2:** Demographic characteristics of female fashion models (n=65) and nonmodels (n=38).

Characteristics		Model, n (%)	Nonmodel, n (%)
**Race/ethnicity**			
	Caucasian	44 (67.7)	21 (55.3)
	Asian (including East Indian)	5 (7.7)	7 (18.4)
	Black	3 (4.6)	3 (7.9)
	Hispanic/Latino	1 (1.5)	2 (5.3)
	Mixed race	12 (18.5)	5 (13.1)
**Highest level of education completed**			
	Graduate school	2 (3.1)	7 (18.4)
	University graduate	12 (18.5)	25 (65.8)
	Some university	6 (9.2)	3 (7.9)
	High school/A level/GED^a^	32 (49.2)	3 (7.9)
	Some high school/A level/GED	9 (13.8)	0 (0)
	Less than high school/A level/GED	4 (6.2)	0 (0)

^a^GED: general educational development.

### Somatomap 2D

**Figure 3:**
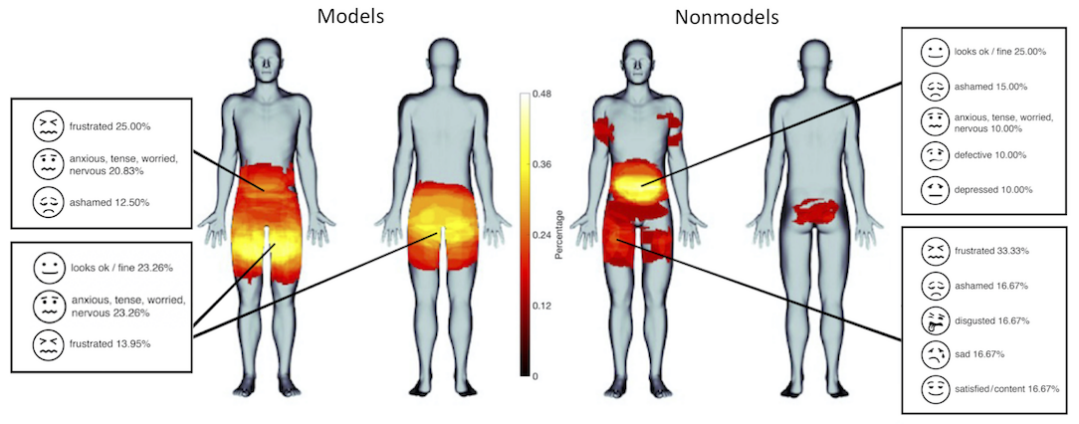
Proportional maps of body image concerns and associated emotions in female fashion models (left) and nonmodels (right).

**Figure 4:**
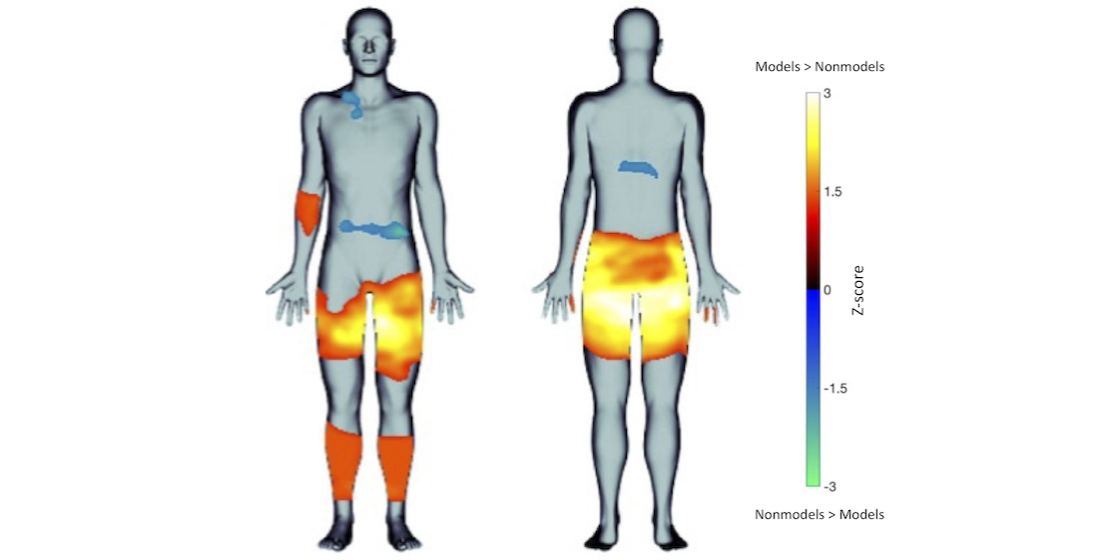
Statistical body map evaluating differences in body image concerns between female fashion models (in warm colors) and nonmodels (in cool colors; statistical threshold: *P*<.05).

Proportional body maps showed that models perceived body concerns in similar as well as distinct areas compared with nonmodels (Figure 3). The number of areas of body concern ranged from 0 to 6 per individual for models (mean 1.2 [SD 0.9]) and from 0 to 4 per individual for nonmodels (mean 1.4 [SD 1.0]), which was not significantly different between groups (t_102_=1.3, *P*=.19). The number of body concern types (eg, acne, bloated, bulgy, and too thin) ranged from 0 to 11 per individual for models (mean 2.4 [SD 2.2]) and from 0 to 12 per individual for nonmodels (mean 2.8 [SD 2.5]), which was also not significantly different between groups (t_102_=0.9, *P*=.35). The number of affective ratings (eg, frustrated and disgusted) ranged from 0 to 7 per individual for models (mean 1.7 [SD 1.4]) and from 0 to 8 per individual for nonmodels (mean 2.0 [SD 1.7]); this too was not significantly different between groups (t_102_=0.9, *P*=.38; see Table 3 for frequency listings of participants endorsing each affective label in each group). The statistical body map analysis revealed several areas that were identified significantly more frequently for each group (*P*<.05) as follows: Models were more concerned with their thighs and buttocks than nonmodels, whereas nonmodels had more frequent concerns about their abdomen than the models (Figure 4). Specifically, 95.8% (46/48) of models who indicated concerns related to the thighs/buttocks described them as oversized (eg, bulgy, too large, protrudes, too fat, too much cellulite, or too much muscularity). Only 4.2% of models (2/48) with concerns related to the thighs/buttocks, described them as too thin and desired more muscle. With respect to nonmodels, 88.2% (15/17) who indicated abdomen concerns described their abdomens as oversized (eg, bloated, too fat, bulgy, too large, protrudes, or too round). Only 11.8% of nonmodels (2/17) with concerns related to the abdomen (eg, acne and flawed) were not size related. Both groups used similar emotions to describe their feelings about their body concerns (Figure 3). The intensity of emotion ratings related to each group’s primary body concern—thighs/buttocks for models (mean 26.81 [SD 29.3]), and abdomen for nonmodels (mean 38.4 [SD 31.4])—was not significantly different (t_24_=–1.30, *P*=.21).

**Table 3:** Frequency and percentage of individual participants endorsing each affective rating per group (models: n=65, nonmodels: n=38).

Affective type		Models endorsing affective rating, n (%)		Nonmodels endorsing affective rating, n (%)
**Negative type**				
	Frustrated		19 (29.2)	7 (18.4)
	Anxious, tense, worried, nervous		18 (27.7)	3 (7.9)
	Other (eg, defeated, annoyed, self-conscious, exhausted, not enough, silly, don’t like)		14 (21.5)	9 (23.7)
	Ashamed		10 (15.4)	9 (23.7)
	Hopeless		5 (7.7)	4 (10.5)
	Sad		4 (6.2)	6 (15.8)
	Disgusted		4 (6.2)	4 (10.5)
	Defective		3 (4.6)	3 (7.9)
	Depressed		2 (3.1)	5 (13.2)
	Fearful		2 (3.1)	1 (2.6)
	Angry		1 (1.5)	2 (5.3)
	Overwhelmed		1 (1.5)	0 (0)
	Lonely		1 (1.5)	0 (0)
	Numb/unreal/dead		0 (0)	1 (2.6)
	Embarrassed		0 (0)	1 (2.6)
**Neutral/positive type**				
	Looks ok/fine		18 (27.7)	13 (34.2)
	Hopeful		8 (12.3)	3 (7.9)
	Satisfied/content		2 (2.1)	4 (10.5)

### Somatomap 3D

A summary of the actual and perceived body area sizes and discrepancies (perceived measure minus actual measurements) is listed in Table 4. The actual measurements for the shoulders and bust were not significantly different between models and nonmodels. However, significant differences were noted with actual sizes of the bicep, waist, hips, thigh girth, and calf girth, with models exhibiting smaller body measurements than nonmodels. Evaluation of discrepancy scores revealed that models and nonmodels were not significantly different for shoulders or thigh girth. However, models were significantly more accurate (ie, had lower discrepancy scores) in perceiving their bust, bicep girth, waist, hips, calf girth, and overall body (scaled average of all seven body scores). Models showed the smallest discrepancy between perceived and actual measurements of their bicep and hips (0.01 and 0.58 cm, respectively), whereas nonmodels showed the smallest discrepancy for the shoulders (0.59 cm). Nonmodels perceived each body area to be slimmer than it actually was (ie, all discrepancy scores were negative). The same was true for models, except for the bicep and hips, although to a lesser degree than nonmodels for most body parts (Table 4).

**Table 4:** Actual and perceived body measurements in female fashion models and nonmodels.

Variable		Nonmodels, mean (SD)	Models, mean (SD)	*P* value^a^	Partial η^2^	Cohen f	*F* (df)^b^		Wilks Λ^b^		*P* value^b^	
**Actual body measurements (cm)**								50.33 (7,91)		0.205		<.001
	Shoulder	32.11 (2.22)	35.18 (2.17)	.10	0.038	0.198						
	Bust	85.76 (6.01)	80.58 (3.99)	.20	0.021	0.144						
	Bicep	28.61 (6.18)	22.37 (2.30)	<.001	0.223	0.537						
	Waist	74.11 (6.45)	64.68 (4.94)	<.001	0.244	0.568						
	Hip	96.11 (7.09)	89.03 (4.65)	<.001	0.101	0.336						
	Thigh girth	46.53 (7.09)	44.58 (3.02)	<.001	0.094	0.322						
	Calf girth	42.08 (6.09)	32.51 (2.75)	<.001	0.450	0.904						
	Scaled body average	0.98 (0.07)	0.99 (0.05)	.98	0.000004	0.002						
**Perceived body measurements (cm)**								4.85 (7,91)		0.382		<.001
	Shoulder	31.55 (1.91)	31.38 (2.05)	.94	0.00006	0.008						
	Bust	79.18 (8.61)	77.32 (8.42)	.09	0.040	0.205						
	Bicep	24.24 (2.80)	22.37 (2.20)	.21	0.019	0.141						
	Waist	62.11 (2.77)	60.31 (2.11)	.53	0.005	0.072						
	Hip	90.97 (5.92)	89.54 (5.73)	.20	0.021	0.147						
	Thigh girth	34.45 (4.60)	31.55 (2.73)	.18	0.027	0.166						
	Calf girth	31.55 (4.12)	29.60 (2.95)	.87	0.0005	0.021						
	Scaled body average	0.98 (0.06)	0.99 (0.04)	<.001	0.145	0.413						
**Differences between actual and perceived measurements (cm)**								21.03 (7,91)		0.205		<.001
	Shoulder	–0.59 (2.58)	–3.83 (3.05)	.19	0.023	0.155						
	Bust	–6.54 (8.12)	–3.23 (8.75)	.03	0.060	0.253						
	Bicep	–4.34 (6.97)	0.01 (2.76)	<.001	0.218	0.529						
	Waist	–11.91 (6.27)	–4.30 (5.20)	<.001	0.166	0.447						
	Hip	–5.04 (8.46)	0.58 (6.13)	<.001	0.092	0.317						
	Thigh girth	–12.05 (9.07)	–12.95 (3.32)	.19	0.024	0.158						
	Calf girth	–10.53 (8.25)	–2.86 (3.54)	<.001	0.336	0.711						
	Scaled body average	–0.62 (0.42)	–0.046 (0.43)	<.001	0.162	0.440						

^a^*P* values corrected for multiple comparisons using the Benjamini-Hochberg procedure.

^b^Measured using multivariate analysis of covariance.

### Somatomap Usability Assessment

A total of 36 nonmodels and 65 models completed the usability rating questionnaire immediately after using the 3D portion of Somatomap (Table 5). Overall, participants found the map easy to use (score for models: 8.4/10; score for nonmodels: 8.5/10). Both groups reported that the final avatar was relatively close to their actual perceived body size (models: 72.7%, nonmodels: 75.2%), but they only identified with the original avatar at a moderate level (score for models: 4.9/10, score for nonmodels: 5.6/10). The groups differed only in terms of enjoyment, with nonmodels reporting higher levels of enjoyment with their experience using the app than models (score for models: 5.9/10, score for nonmodels: 7.4/10; t_70_=2.91, *P*=.002). Qualitatively, participants commented that they “liked best” that the app was “easy to use,” “fun to use,” and “easy to control.” Several reported that they enjoyed the visual nature of the tool, but also preferred having additional options for changing hair and skin type as well as control over more body areas.

**Table 5:** Somatomap usability assessment results.

Usability questions	Models (n=65), mean (SD)	Nonmodels (n=36^a^), mean (SD)
1. How easy was this app to use? (1 - extremely difficult to 10 - extremely easy) Please explain.	8.4 (2.42)	8.5 (1.76)
2. What was your experience using this app? (1 - extremely frustrating to 10 - extremely enjoyable) Please explain.	5.9 (2.34^b^)	7.4 (2.43^b^)
3. How much did you identify with the original avatar? (0 - not at all to 10 - completely) Please explain.	4.9 (2.75)	5.6 (2.34)
4. How closely did the final avatar you created reflect your body? (0% - not at all to 100% - completely) Please explain.	72.7 (20.17)	75.2 (17.09)

^a^Two participants were unable to complete the user experience questionnaire because they needed to get to work; therefore, n=36 instead of 38.

^b^*P*=.002.

## Discussion

In this study, we developed and pilot tested Somatomap, a novel mobile tool for assessing body image perception in both 2D and 3D. We tested this tool in female fashion models, who we hypothesized, given their profession, would have greater expertise with and therefore accuracy in estimating their body shape and size relative to female nonmodels. Both groups reported body concerns but in different areas, with models more concerned with the thighs/buttocks and nonmodels, with the abdomen/waist. Models were more accurate at estimating their overall body size, whereas nonmodels tended to underestimate the size of individual body parts, showing greater discrepancy scores for the bust, biceps, waist, hips, and calves, but not shoulders and thighs. Both groups reported high ease-of-use scores and felt that the resulting 3D avatar closely resembled their actual body, suggesting good usability experience with this tool. Overall, these pilot results suggest that Somatomap is feasible to use and capable of providing unique insight into how humans perceive and represent the visual characteristics of their body.

Body image perception is an inherently subjective phenomenon that is challenging to measure directly. To date, the standard methods for assessing body image perception in clinical settings have relied on verbal interviews, paper-based manikins, and still photographs [34-36,47]. Advantages of Somatomap 2D over lengthy paper-based or verbal interviews include the ease of visually representing one or more areas of appearance concerns, the ability to individually describe types of concerns and associated emotions for each area, and the ability to perform statistical body map comparisons to quantify and visually represent differences in areas of body concern. Somatomap 3D, as reported by participants in this study, is easy to use and able to closely approximate individual body types. This suggests good flexibility to visually represent how users perceive themselves, which is an advantage over other visually based tools that use fixed bodies to select from. Computerized assessments exist that assess separate characteristics of body image such as overall size or shape [37-40], but they do not provide the same individual body-part flexibility as Somatomap 3D. Another advantage of Somatomap 2D and 3D is the level of detail of information that may be obtained via assessments of individual body areas. Instead of merely assessing body image concern or dissatisfaction as a whole or overall, Somatomap 2D allows individuals to specify unique details associated with each area of concern, such as the physical characteristics and the associated affective experiences. Somatomap 3D, in combination with physical measurements, allows for quantification of perception discrepancy for *individual* body areas, rather than only for the body shape as a whole, as is common with other existing assessments (such as the Stunkard Figure Rating Scale).

We created Somatomap in an effort to achieve, as objectively as possible, an accurate digital snapshot of body image concerns, a quantification of perceptual accuracy between one’s internalized and actual body form at the level of individual body parts, and an ability to relate the two. Statistical body maps in Somatomap 2D identified female fashion models as having significantly more concerns about the thighs (especially the inner thigh) being too large compared to the nonmodels. This particular body concern may reflect a trend toward the desirability of having a “thigh gap,” that is, a gap or space between the thighs when standing upright with the feet together. For example, a 2015 online survey of 500 UK females found that 40% of women aged 16-65 years felt that they would feel more confident if they had a “thigh gap” [48]. Results from Somatomap 3D showed that both groups underestimated individual body areas, in agreement with studies suggesting that women tend to underestimate their body size in the general population [49], but not with studies suggesting women in the general population may overestimate their body size [12,13,50]. Models were also significantly more accurate at estimating their overall body size than nonmodels and were more accurate at estimating the size of their bust, biceps, waist, hips, and calves. Interestingly, when examining body discrepancy scores between groups in Somatomap 3D, the thighs were one of only two areas where the groups did not differ in their estimation ability, yet they were an area for which models endorsed significantly more frequent concerns than nonmodels in Somatomap 2D. These initial results suggest that in combination, Somatomap 2D and 3D have the ability to detect differences in body image perception for the same body part that is distinct.

These results in models and nonmodels may provide partial support for the social norm hypothesis, which states that judgments of body size/weight are influenced by visual proximity to different body types [51]. Given the ongoing obesity epidemic in nondeveloping and developing countries, this suggests that a recalibration of body sizes is underway, leading to a perception that larger body sizes are “normal.” Thus, according to this hypothesis, the nonmodels may have calibrated their body perception by comparing themselves mainly to the general UK population (ie, 61% of which are overweight or obese [52]), whereas the models may have calibrated their body perception by comparing themselves to their general peers (other slimmer-than-average fashion models). Despite a higher BMI than models, the nonmodel sample recruited in this study (in the healthy range on an average) exhibited a lower average BMI than the overall general UK population norms would suggest. This made them a fairly good comparisons for the models, but might have also potentially resulted in an underestimation of body image discrepancies for general UK female nonmodel samples overall.

By facilitating the accurate measurement of attitudinal and perceptual aspects of body image disturbance, the Somatomap tool may allow for subsequent characterization of the underlying neural mechanisms in clinical and nonclinical conditions. For example, as the pilot results suggest, it is plausible that this tool should be sensitive to detecting overestimation discrepancies of specific body areas (eg, waist, hips, and bust) that have been noted in individuals with anorexia nervosa [11-13] and others that have been observed in body dysmorphic disorder [17-19]. Pairing this tool with neurobiological measures such as functional magnetic resonance imaging or electroencephalography might help elucidate if and how previously described abnormalities of cortical visual systems [20-22] could be linked to misestimations of specific body areas, which, in turn, may contribute to body image disturbance in anorexia nervosa and body dysmorphic disorder. At the clinical level, it remains to be seen whether this tool can effectively and reliably measure distortions of body image perception in these disorders, and further studies will be required to determine the viability of this approach.

By providing better insights into the perceptual mechanisms, Somatomap may assist in the effort to uncover latent factors underlying body image disturbance in various psychiatric illnesses, reveal important information about illness course, and possibly contribute to the development of novel treatments. When developing Somatomap, we aimed to generate a mobile tool capable of deployment over a broad range of devices, physical locations, and settings (ie, research and clinical). The cross-platform compatibility and HIPAA-compliant encryption (via Chorus), along with the estimation that 80% of adults will own a smartphone by 2020 [53], represents a significant first step in this direction. Longitudinal deployment of this tool may assist clinicians in detecting the response of body image disturbance to existing clinical interventions and in the longitudinal tracking of illness course. Although speculative, it seems possible that this tool could also contribute to the development of novel interventions for body image disturbance. For example, virtual reality has shown therapeutic potential in helping recalibrate body perception discrepancies in anorexia nervosa. A recent virtual reality study [54] modulated the sense of ownership of a virtual body avatar using visuotactile stimulation and showed that it reduced overestimations of the abdomen for up to several hours. This kind of perceptual retraining might also be investigated with Somatomap using mobile devices, particularly in settings in which identification with the avatar can be maximized, and when access to virtual reality or other specialized equipment is difficult. Therefore, the Somatomap tool might potentially have a clinical impact in patients, such as deployment of the tool to assist clinicians in detecting the response to treatments targeting body image disturbance; longitudinal tracking of naturalistic illness course (ie, for remote surveillance of potential relapse or “flare ups” of body image disturbance); or integration as a component of novel perceptual retraining interventions, particularly in remote settings when access to specialized equipment such as virtual reality may be limited.

This study has several limitations. First, usability data were obtained from participants after using the 3D assessment portion of the tool. We did not collect separate usability data for the 2D assessment. Second, data collection occurred in a relatively small sample of women from the United Kingdom. Obtaining measures, and eventually norms, across a greater variety of different racial/ethnic, socioeconomic groups, and sexual/gender categories will be important for determining the generalizability of this approach to global populations. Third, the 2D manikin consisted of an androgynous figure, and it is unclear if a sex-specific figure would alter the type of assessments provided. However, having a consistently sized 2D model enabled us to perform statistical analyses across subjects more easily. Fourth, identification with the 3D avatar (before manipulation) was in the moderate range, and while it improved a lot after the final manipulation, it was not at the highest possible limit. Possible changes to further improve avatar identification might include offering more customizability of different features beyond the hair and skin color options currently supported in the generic avatar, increasing the number of areas that can be modified (ie, beyond the seven presented here), adding new body modification parameters such as height/length (ie, beyond the girth/width modification ability presented here), and improving avatar personalization, as it was recently noted that “personalized avatars significantly increase body ownership, presence, and dominance compared to their generic counterparts” [55]. As this was a pilot feasibility study, we did not examine test-retest reliability or formally compare results to existing body image assessment tools (such as the Stunkard Figure Rating Scale) to evaluate construct validity. We anticipate performing such evaluations after revising Somatomap on basis of the user experience data collected in this study. Finally, we did not perform clinical diagnostic evaluations or have access to medical records to determine the presence of eating disorders, body dysmorphic disorder, or other psychiatric disorders that could affect body perception in either sample.

Overall, these pilot results suggest that Somatomap is feasible to use and capable of providing unique insights into how humans perceive and represent the visual and size/shape characteristics of their body. Its advantages over commonly used tools include mobility; ease of use; customizable avatars that can flexibly represent users’ bodies with a variety of body shapes and sizes; and most of all, the ability to visualize and statistically quantify body image perception at the level of both individual body concerns (Somatomap 2D) and perceptions of individual body part size and shape (Somatomap 3D). Future clinical applications of this tool could include investigations of appearance concerns and body perception in disorders involving body image, such as eating disorders and body dysmorphic disorder. This potentially could be used both cross-sectionally as well as longitudinally to follow illness trajectory and changes over time with treatment.

## Abbreviations

BMI: body mass index
